# The Methylome of Vertebrate Sex Chromosomes

**DOI:** 10.3390/genes9050230

**Published:** 2018-05-01

**Authors:** Shafagh A. Waters, Alexander Capraro, Kim L. McIntyre, Jennifer A. Marshall Graves, Paul D. Waters

**Affiliations:** 1 School of Biotechnology & Biomolecular Sciences, Faculty of Science, University of New South Wales, Sydney, NSW 2052, Australia; shafagh.waters@unsw.edu.au (S.A.W.); a.capraro@unsw.edu.au (A.C.); kim.mcintyre@student.unsw.edu.au (K.L.M.); 2 School of Women’s and Children’s Health, Faculty of Medicine, University of New South Wales, Sydney, NSW 2052, Australia; 3 School of Life Science, La Trobe University, Melbourne VIC 3086, Australia; j.graves@latrobe.edu.au

**Keywords:** X chromosome inactivation, dosage compensation, DNA methylation, 5mC, mammal, bird, sex chromosome

## Abstract

DNA methylation is a key epigenetic modification in vertebrate genomes known to be involved in the regulation of gene expression, X chromosome inactivation, genomic imprinting, chromatin structure, and control of transposable elements. DNA methylation is common to all eukaryote genomes, but we still lack a complete understanding of the variation in DNA methylation patterns on sex chromosomes and between the sexes in diverse species. To better understand sex chromosome DNA methylation patterns between different amniote vertebrates, we review literature that has analyzed the genome-wide distribution of DNA methylation in mammals and birds. In each system, we focus on DNA methylation patterns on the autosomes versus the sex chromosomes.

## 1. Introduction

Genetic sex determination is often accompanied by the presence of differentiated sex chromosomes. Within mammals, therians (eutherians and marsupials) typically have an XX female/XY male sex chromosome system. A male dominant testis-determining factor (TDF) is located on the Y chromosome and was identified as the *SRY* gene in humans and mice [1,2]. Its presence on the Y chromosome also in marsupials means that it must have arisen in the therian ancestor about 150 million years ago [3]. 

**Figure 1:**
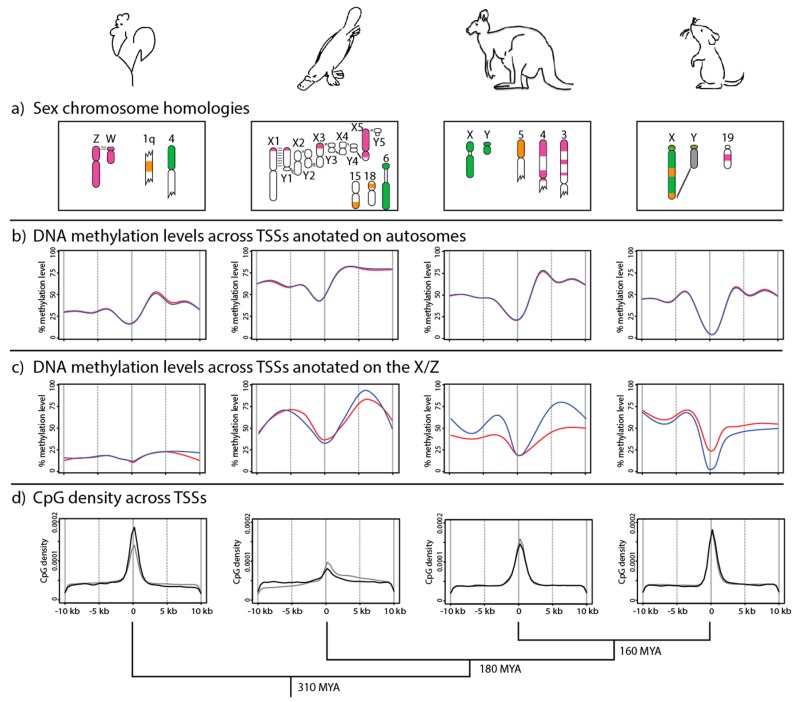
Phylogenetic relationships and DNA methylation profiles of amniote vertebrates. (**a**) Sex chromosome homologies with the therian mammal X conserved region (green) and the eutherian X added region (orange), which is autosomal in marsupials. The platypus X chromosomes and the chicken Z chromosome (purple) share extensive homology, especially X5 which is almost entirely homologous to the chicken Z. However, despite this homology, these sex chromosome systems have independently evolved. (**b**) Metagene analysis (adapted from [9]) of DNA methylation levels across transcription start sites (TSSs) of expressed autosomal genes in males (blue) and females (red). The absolute level of DNA methylation varies between species, but there is a conserved pattern of low DNA methylation flanked by high methylation (especially downstream of TSSs in marsupials, monotremes, and birds). (**c**) Metagene analysis (adapted from [9]) of DNA methylation levels across TSSs of all genes annotated on the chicken Z and platypus Xs, and genes subject to X chromosome inactivation (XCI) in marsupials and eutherians, in males (blue) and females (red). In chicken and platypus, the methylation patterns do not vary between sexes. However, in female marsupials, DNA methylation flanking TSSs is reduced relative to males, whereas, in female eutherians, DNA methylation at TSSs is increased relative to males. (**d**) Density of CpG dinucleotides relative to annotated TSSs on autosomes (black) and the X/Z chromosome (grey). In all species, CpG densities peak at TSSs, with X chromosomes mirroring the autosomes in therian mammals. The change in X chromosome versus autosome density in platypus likely results from the relatively few genes anchored to the Xs. The chicken Z chromosome has a reduced density of CpGs at TSSs compared to the autosomes, which could result from a diminished function for DNA methylation in Z chromosome gene regulation (see text).

The gene content of the long arm and proximal short arm of the human X chromosome is conserved on the marsupial X, defining an X-conserved region (XCR). The remainder of the human X is autosomal in marsupials (and is separate from the XCR in other vertebrates), defining an X-added region (XAR) that was translocated to the sex chromosomes in the eutherian mammal ancestor (reviewed in [4]) (Figure 1a).

Monotreme mammals have an unusual sex chromosome system that shares no homology with the therian XY pair, which is instead present as an autosome. Platypuses have five X chromosomes, each present in two copies in females and one copy in males. In males, there are five Y chromosomes that pair with the X chromosome to form a meiotic chain at meiosis. The monotreme sex chromosomes share considerable homology with the ZZ male/ZW female sex chromosome system of birds [5,6,7]; however, the variety of sex chromosome systems in birds and reptiles makes identity by descent unlikely. Therefore, the sex chromosomes of therians, monotremes, and birds evidently all evolved independently of each other [8] (Figure 1a).

In this review, we outline the processes of sex chromosome evolution and dosage compensation and relate them to the DNA methylation patterns on different sex chromosome systems. To understand this relationship and its implications for a potential role in gene silencing, we review DNA methylation patterns from birds through to mice. We reveal that the distribution of CpG dinucleotides on autosomes is consistent between species and discuss the implications of dissimilar DNA methylation patterns in each of the different sex chromosome systems.

## 2. Sex Chromosome Evolution

The human X chromosome bears 843 protein-coding genes [10], whereas the male-specific region of the human Y bears only approximately 25 unique protein-coding genes, with many present in multiple copies [11,12,13,14]. Although the X and Y chromosomes differ greatly in both structure and gene content, most of the genes on the Y chromosome have a partner on the X. It is now widely accepted that they evolved from a homologous pair of autosomes, after the emergence of *SRY*, via degeneration of the Y chromosome [12,15]. This is consistent with a model in which the acquisition of a novel TDF was followed by the nearby accumulation of alleles that were beneficial to males (in linkage disequilibrium) on the proto-Y chromosome, increasing the likelihood of being inherited in males. Recombination between the X and Y was suppressed across the male beneficial alleles, possibly by multiple inversions on the Y [16], defining the first multigene region of the Y chromosome that was male-specific. 

Suppression of recombination between the X and Y signaled the demise of genes on the Y. Selection no longer acted on individual loci, but rather on the male-specific region of the Y as a single locus. Deleterious mutations were fixed in the population by the loss of Y chromosomes that were free of deleterious mutations (Muller’s ratchet) or by selection at linked loci (genetic hitchhiking/ background selection) [12,15]. This led to Y chromosome degeneration and loss of functional genes that were insensitive (or almost insensitive) to haploinsufficiency. On the therian Y, all but a handful of genes were lost: only 20 genes from the ancestral proto-Y remain on the Y chromosome in at least one therian representative [12,13,14]. In contrast, gene content of the X has remained largely unchanged. All that remains of the once extensive homology between the X and Y is a small pseudoautosomal region (PAR), within which recombination still occurs. In humans, outside of the PAR, there are approximately 20 genes with a copy on both the X and Y. 

This process of sex chromosome evolution has resulted in differentiated sex chromosomes in diverse vertebrate lineages. A presumably analogous process of W chromosome degeneration led to the evolution of the bird ZW system [17]. The first XY pair in the monotreme ancestor (represented by platypus X5 and Y5) evolved after the acquisition of a sex-determining allele—potentially *AMH* on Y5 [14]. The multiple sex chromosome system evolved via subsequent serial translocations with autosomes [18]. There are alternative models for sex chromosome evolution, even in vertebrate lineages. For example, in some cichlid fishes, a B chromosome, derived from the Z, was recruited into a female-specific W chromosome role [19]. However, these examples are not common.

## 3. Dosage Compensation: A Shared Problem

Degradation of the Y chromosome and loss and inactivation, or specialization, of Y genes has left genes on the X chromosome as single-copy in males. Their expression relative to autosomal genes with which they interact is therefore expected to be halved [20]. Dosage compensation can mitigate this difference in several different ways, including male-specific upregulation of X genes, as in Drosophila [21], or inactivation of one X in females, as in eutherian mammals [22].

The initial step in the evolution of dosage compensation in mammals, as proposed by Ohno in 1967 [23], was a two-fold increase in the expression of X-borne genes in males to maintain parity with the autosomes. The overexpression of X genes carried through to females, producing a deleterious excess of X chromosome product in females. This was countered by a mechanism to inactivate one of the two X chromosomes in the somatic cells of females (X chromosome inactivation—XCI). 

This model for the evolution of XCI has been challenged by global measures of X chromosome transcriptional output (see [20]), with perhaps only a subset of dosage-sensitive genes responsible for driving the evolution of XCI [24,25]. In eutherian mammals, the transcript levels from the X chromosomes are balanced between males and females, but the global transcript levels of X genes are lower (by almost half) than the autosomal average in both sexes. However, upregulation of genes on the X chromosome (to autosomal levels) was observed in male opossum [20], consistent with Ohno’s classic hypothesis.

In different vertebrate lineages, a number of different processes compensate for X chromosome imbalance, ranging from chromosome wide strategies such as XCI, to regional or gene-specific mechanisms (reviewed in [25]). These mechanisms have arisen on independently evolved sex chromosome systems, so cannot be identical by descent. However, they share common features that display parallels with other gene silencing phenomena, e.g., genomic imprinting. The various silencing strategies must have been exapted from an ancient epigenetic toolbox shared across eukaryotes. Dosage compensation mechanisms are best understood in eutherian mammals, in which one whole (or nearly whole) X chromosome is epigenetically silenced (i.e., XCI) in the somatic cells of females. In other amniote vertebrates, global inactivation appears not to occur; in marsupials, monotremes, and birds, sex chromosome silencing is regional (or locus specific) and incomplete at best [26,27,28]. A completely different dosage compensation mechanism, mediated by microRNA, has been proposed for chicken [29]. 

## 4. X Chromosome Inactivation in Mammals

X chromosome inactivation has become a paradigm for the epigenetic silencing of a whole chromosome, but there are major differences in its mechanism in marsupials and eutherians. Although the sex chromosomes of marsupials and eutherians share a common origin, and despite the similarities of XCI in the two systems, the mechanism that mediates XCI evolved independently. In both mammal groups, XCI is mediated by X-borne long noncoding RNAs (lncRNAs). In eutherians, this is *XIST* (X-inactive specific transcript) [30] and, in marsupials, it is a completely different noncoding RNA called *RSX* (RNA on the silent X) [31]. The epigenetic changes associated with XCI are initiated after expression of *XIST* or *RSX* RNA, which in each case spread in *cis* to coat the X chromosome to be inactivated.

In eutherians, coating of the X with *XIST* RNA recruits the chromatin silencing machinery [32,33,34]. The inactive X accumulates repressive histone modifications (e.g., H3K27me3 and H3K9me2) and loses active modifications such as H4Kac (reviewed in [35]). DNA methylation is a late, stabilizing modification thought to “lock in” the inactive state. In humans, the inactive X is not completely silenced, 80 out of 639 genes escape (or mostly escape) XCI, and 49 out of 639 genes variably escape XCI [36]. These genes are primarily located on the short arm of the X [37], reflecting incomplete inactivation of the recently added region (XAR). Escape from inactivation of genes in this region is also common on the X chromosomes of other eutherian mammals, such as elephants (six out of 11 genes escape) [38]. However, only 19 X genes escape XCI in the mouse brain, and just seven escape in the spleen [39].

The inactive X in marsupials accumulates some of the same epigenetic modifications as in humans (e.g., H3K27me3 and H3K9me2). It is also depleted of histone acetylation that is associated with active chromatin [40]. However, several modifications associated with the inactive X in humans are absent from the marsupial inactive X (H4K20me1) or are restricted to specific stages of the cell cycle (e.g., H3K27me3 and H3K9me2). The marsupial inactive X also associates with repressive histone modifications (H3K9me3 and H4K20me3) that do not associate with the inactive X in eutherians [41,42,43]. These data demonstrate that marsupials and eutherians share some, but not all, epigenetic signatures on their inactive X chromosomes. 

DNA methylation at CpG dinucleotides is a well-characterized epigenetic modification. In eukaryotes, the silent chromatin state is locked in by DNA methylation (reviewed in [44]). Although its role in gene silencing appears to be widely conserved, the evolution of DNA methylation patterns on sex chromosomes is poorly understood. The importance of DNA methylation to eutherian XCI has long been appreciated [45], whereas a potential role in marsupial XCI (or dosage compensation in other vertebrates) has remained obscure (reviewed in [46]).

## 5. DNA Methylation

Forty years of research support the association between cytosine DNA methylation (5-methylcytosine—5mC) and gene expression originally suggested by Holliday and Pugh [47]. It is involved in a diverse range of cellular functions, including tissue-specific gene expression, cell differentiation, development, genomic imprinting, and XCI. The central hypothesis is that DNA methylation acts as a physical barrier modulating transcription factor and transcriptional machinery access to chromatin [48]. DNA methylation is an unusually stable epigenetic modification in somatic cells, essentially because 5mC is copied to the nascent strand during DNA replication [49], ensuring somatic heritability. 

**Figure 2:**
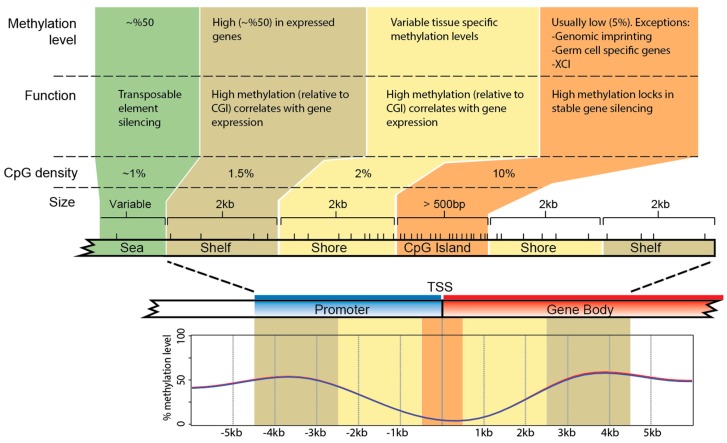
Typical CpG island structure in the promoter (blue) and into the gene body (red) of a eutherian gene. DNA methylation levels are shown for expressed genes, along with how methylation levels correlate with gene expression [53,54,55]. CpG islands (CGIs) are regions of dense CpGs, within a window of at least 500 bp, that generally exhibit low methylation in expressed genes [59,60]. CpG shores and shelves contain fewer CpG sites. Shores have more dynamic DNA methylation levels depending on tissue type [53,59,60]. There is high DNA methylation in seas, which is thought to silence transposable elements [61]. The DNA methylation level (from Figure 1b) across TSSs of expressed genes in mouse liver is shown below the gene. Dashes immediately above CGIs, shores, and shelves represent CpG dinucleotides, with highest density in the CGI near the TSS. Statements about the upstream shore and shelf apply also to gene body shore and shelf.

CpG dinucleotides are present in the mammal genome at only one-fifth of their expected level. Depletion is due to the hyper-mutability (approx. 11-fold) of cytosine when methylated [50]. These relatively rare CpGs (on average 1 per 100 bp) are non-randomly distributed across the genome, clustering in regions termed CpG islands (CGIs) (reviewed in [51]). CpG islands predominantly exhibit low methylation levels in most tissue types [52,53]. DNA methylation levels are more dynamic in regions up- and downstream of the islands, that is, CGI shores (0–2 kb up- and downstream of CGIs) and CGI shelves (2–4 kb up- and downstream of CGIs) [54,55] (Figure 2).

Mammalian methylomes are established in early development by two DNA methyltransferases (DNMT3A and DNMT3B) [56]. CpG island promoters are protected from de novo methylation through various developmental stages [57]. The exceptions to this are the CpG island promoters on the inactive X chromosome in female somatic cells [58]. 

## 6. DNA Methylation and Eutherian X Chromosome Inactivation 

The silencing role of DNA methylation on the inactive X in eutherian mammals was demonstrated over three decades ago. Treatment of mice and human cell hybrids with 5-azacytidine (to deplete 5mC) led to reactivation of alleles on the inactive X chromosome [45,62,63]. In early genome-wide studies, the active X was shown to be more methylated than the inactive X chromosome, as a consequence of concentrated DNA methylation in gene bodies [64]. Utilizing methyl DNA immunoprecipitation (meDIP) microarray analyses [65,66], DNA methylation was shown to be high at the promoters of X-borne genes subject to inactivation, whereas the promoters of genes that escape X inactivation were hypomethylated. In addition, DNA methylation at X-borne promoters without an associated CGI did not correlate with the X inactivation status [65], and genes with a varied silencing status between individuals had intermediate levels of DNA methylation [66].

Whole-genome bisulfite sequencing in humans and mice supported these studies, revealing patterns of DNA methylation that predicted the escape of genes from X chromosome inactivation [67,68]. These studies examined DNA methylation in both the CG (mGG) and non-CG (mCH) context, revealing that escaper genes have reduced promoter mCG. An increase in intragenic mCH levels was also observed, the functional importance of which remains obscure.

The same themes of high DNA methylation at the promoters of genes subject to inactivation and low DNA methylation at the promoters of genes escaping inactivation are evident in the most recent studies [9,69]. However, finer details also emerged. For example, gene body DNA methylation showed tissue-specific profiles with no clear relationship between overall DNA methylation and expression levels [69], although low DNA methylation in the first exon correlated with high gene expression [70]. The problem remains that there is a lack of repeated detailed examination of DNA methylation outside of eutherians (mouse and human).

## 7. DNA Methylation and Marsupial X Chromosome Inactivation

The role of DNA methylation in marsupial XCI has long been debated (reviewed in [46]). Single-gene analysis of 27 promoters (in different marsupial species and tissues) revealed no differential methylation between the active and inactive X alleles [71,72,73,74]. These observations supported a general consensus that DNA methylation has no role in marsupial X inactivation.

However, hypomethylation of the inactive X (relative to the active X) in marsupials was reflected in an early study of the *Hprt* gene in common wallaroo [75]. CpGs in the intragenic regions of *Hprt* had reduced DNA methylation on the inactive X. Later, immunofluorescence studies revealed that the inactive X was globally hypomethylated compared to the active X in both American and Australian marsupial species [42,76]. This is inconsistent with observations in eutherians that hypermethylation at promoters correlates with transcriptionally silenced genes. 

How can these conclusions be reconciled? Recently, we described reduced representation bisulfite sequencing (RRBS) [9] from male and female liver DNA of mouse, grey short-tailed opossum (*Monodelphis domestica*), platypus, and chicken. DNA methylation values were calculated for each CpG (with a read depth of >8 in both sexes) as a percentage of reads that were non-bisulfite-converted cytosines (methylated) versus total read depth. CpG position was determined relative to annotated TSSs in each species. These data were used for a metagene analysis across TSSs of genes on sex chromosomes and autosomes.

Our observations confirmed the apparent dichotomy between whole X chromosome and gene-specific DNA methylation patterns. In grey short-tailed opossum, the X chromosome was globally hypomethylated in females compared to males, the difference attributed to the possession of an inactive X chromosome in females. DNA methylation levels were equivalent between the sexes at annotated TSSs on the X chromosome, however the regions flanking TSSs were hypomethylated in females [9]. This pattern of methylation on the marsupial X contrasts with that on the eutherian X chromosome. 

These patterns can be compared with methylation patterns of sex chromosomes outside of therian mammals (see below).

## 8. DNA Methylation Patterns in Monotremes and Birds

There is evidence for partial silencing of a limited number of loci on the X chromosomes of female platypus and Z chromosome of male chicken [27]. However, more recent observations do not support global silencing of the Z-borne loci in male chicken [28]. Rather, there is evidence that microRNAs could modulate the expression of dosage-sensitive Z genes [29]. In both chicken and platypus, no differential DNA methylation (between males and females) was detected on either the autosomes (Figure 1b) or the sex chromosomes (Figure 1c) [9]. Autosomal TSSs displayed a pattern of relatively low DNA methylation that was flanked by higher methylation (especially downstream) (Figure 1b). This pattern was mirrored across autosomal TSSs in marsupials and eutherians (see below). In chicken, the absolute level of DNA methylation across TSSs was up to 2-fold lower than in the other species, possibly a reflection of the reduced genome-wide DNA methylation levels [77].

Despite the homology shared by the chicken Z and platypus Xs (especially X5), these independently evolved sex chromosome systems have very different TSS DNA methylation landscapes. In chicken, there was uniformly low DNA methylation across TSSs on the Z in both sexes (Figure 1c). However, in both male and female at TSSs on the platypus Xs, DNA methylation was similar to the autosomal pattern, with high DNA methylation flanking lower DNA methylation (Figure 1c). The lack of differential methylation patterns between the sexes suggests that robust transcriptional silencing mediated by DNA methylation is not required for chicken and platypus dosage compensation. Each of the four species examined had strikingly different DNA methylation profiles across X- (or Z-) borne TSSs [9].

## 9. Evolution of DNA Methylation Patterns on Sex Chromosomes

The non-homologous sex chromosome systems of birds, monotremes, and therian mammals [7] must have independently evolved dosage compensation systems. Outside of therian mammals, we know little of the specific molecular basis of sex chromosome dosage compensation in amniote vertebrates [20,27,28,29]. The different DNA methylation landscapes across TSSs of genes on the X/Z in these groups might offer clues to the molecular basis of these dosage compensation schemes [9] and sex chromosome biology more broadly. 

The variation in the DNA methylation landscape on the sex chromosomes of different species is unlikely to be the result of differences in genomic CpG distribution. CpG distribution is similar in chicken, platypus, opossum, and mouse, each of which shows a higher density of CpGs at annotated TSSs (Figure 1d). Higher CpG density at TSSs is evident on both the autosomes and the sex chromosomes, although, in chicken, the CpG density at TSSs on the Z chromosome is lower than on the autosomes. In platypus, the autosomal and sex chromosome profiles of CpG density differ somewhat, but this might simply reflect the incomplete genome assembly and relatively few genes anchored to the Xs. Given the consistency of CpG density (relative to TSSs) between species and between sex chromosomes and autosomes, differences in CpG methylation profiles are likely to reflect different epigenetic strategies for the regulation of gene expression in these vertebrate groups. 

Although the absolute levels of DNA methylation at autosomal TSSs are different between species, the methylation profiles are similar, in that regions with low DNA methylation are flanked by regions with higher methylation in each species [9] (Figure 1b). This is consistent with a conserved role for DNA methylation in the regulation of gene expression (reviewed in [78]). 

This conserved autosomal landscape of DNA methylation is not observed in sex chromosomes. In chicken, universally low DNA methylation on the Z in both sexes suggests that the epigenetic architecture of the Z is fundamentally different to that of the autosomes, and that expression of genes on sex chromosomes must be modulated by other mechanisms. Absence of a role for DNA methylation in regulating Z chromosome gene expression could explain the reduced density of CpGs at Z-borne TSSs (Figure 1d). Without DNA methylation regulating gene expression, there would be no pressure to maintain high CpG density at promoters via purifying selection. The recent observation that microRNAs might regulate Z chromosome gene expression in a sex-specific manner is consistent with this DNA methylation pattern [29].

In platypus, there are five X chromosomes that are minimally paired with Y partners. The fragmented assembly and lack of anchoring scaffolds to chromosomes makes it impracticable to analyze X chromosomes individually, so genes on all five X chromosomes were analyzed together. No differential DNA methylation was observed between the sexes across the TSSs (Figure 1c) [9]. Unlike the chicken Z, the methylation profile on the monotreme Xs more closely mirrored the autosomal pattern, suggesting that expression of genes on the Xs and autosomes are regulated in similar ways in monotremes. It remains possible that individual X chromosomes have sex-specific differential DNA methylation, a possibility that could be tested after improvement of the assembly.

There are many studies of the role of DNA methylation in eutherian XCI (mostly human and mouse) [65,66,67,69]. These studies all indicate that high DNA methylation is a common feature in the promoters of genes subject to XCI. This has always conformed to the hypothesis that high DNA methylation correlates with reduced gene expression. 

However, more recent and detailed work on DNA methylation and gene expression has challenged this dogma. It was proposed that the spatial distribution of DNA methylation across promoters, rather than its absolute average level, correlates better with gene expression [53,79,80]. High gene expression is characterized by low DNA methylation at TSSs, flanked by high DNA methylation. In contrast, uniformly high DNA methylation at promoters (as on the inactive X in eutherians) correlates with reduced gene expression—but so does uniformly low DNA methylation. 

A pattern of universally low DNA methylation was observed across TSSs of genes that were subject to XCI in marsupials [9] (Figure 1c). This is the first evidence of non-random differential DNA methylation between the active and inactive X chromosomes in marsupials. Just as uniformly high DNA methylation across TSSs is thought to lock in the silent state of eutherian inactive X, we proposed [9] that uniformly low DNA methylation across TSSs plays an analogous role of maintaining the silent state of the marsupial inactive X. 

The eutherian and marsupial X chromosomes share a common origin, so a common origin for a phenomenon as important as XCI is an intuitive assumption. Indeed, a common origin would explain the overlapping histone code present on the inactive X in each group. However, the assumption of identity by descent of XCI mechanisms fails to address the most fundamental differences between eutherian and marsupial XCI: (1) Independently evolved lncRNAs (*XIST* and *RSX*) mediate XCI in the different groups; (2) Ohno’s classic hypothesis of transcriptional upregulation of the X is observed in marsupials, but not in eutherians; (3) The DNA methylation profiles are different. 

Marsupial XCI is often considered the primitive cousin of eutherian XCI, being seen as parent-specific, leaky, and lacking epigenetic layers that make eutherian XCI so “robust”—even though eutherian XCI is also leaky [37]. We propose that marsupial and eutherian XCI are systems of equal complexity that evolved largely independently from a therian ancestor with a “primitive” version of X inactivation. Under this model, the fundamental differences between marsupial and eutherian XCI must have been established after the marsupial/eutherian split, so major features of modern XCI were absent at the time of this divergence. 

The nature of the primitive X inactivation system in a common therian ancestor is of great interest. In the absence of a chromosome-wide silencing strategy, X inactivation was probably incomplete and/or locus-specific. The overlapping histone codes of the eutherian and marsupial inactive X probably reflect this early, relatively simple, shared system. The long noncoding RNAs *XIST* and *RSX* that now provide coordinated XCI control evolved independently in eutherian and marsupial common ancestors. These ancestors likely had pervasive low-level transcription throughout their genomes (like modern humans), providing many ncRNAs as starting material to “choose” from [25]. Additional layers of different epigenetic complexities (DNA methylation, upregulation of the active X) were then independently established in each lineage. 

## 10. Summary

DNA methylation is an important epigenetic mark that has long been recognized for its silencing role in X chromosome inactivation in eutherian mammals. However, in birds and monotremes, there is no evidence for differential DNA methylation between males and females on the sex chromosomes, indicating that DNA methylation is unlikely to play any role in dosage compensation gene silencing in these lineages. In chicken, the differential DNA methylation between the sex chromosomes and autosomes provides evidence that the Z chromosome is epigenetically unique. In all mammals (monotremes, marsupials, and eutherians), genes on the X in males (i.e., an active X) have DNA methylation profiles that mirror those of the autosomes. This suggests that, unlike birds, gene expression on sex chromosomes and autosomes is not modulated by fundamentally different strategies. Finally, genes on the marsupial and eutherian inactive X chromosomes have distinct DNA methylation patterns, indicating that this epigenetic layer of sex chromosome regulation was established independently (like many other features) in each lineage after the divergence of the two infraclasses of therian mammals.
